# Submesoscale inverse energy cascade enhances Southern Ocean eddy heat transport

**DOI:** 10.1038/s41467-023-36991-2

**Published:** 2023-03-11

**Authors:** Zhiwei Zhang, Yuelin Liu, Bo Qiu, Yiyong Luo, Wenju Cai, Qingguo Yuan, Yinxing Liu, Hong Zhang, Hailong Liu, Mingfang Miao, Jinchao Zhang, Wei Zhao, Jiwei Tian

**Affiliations:** 1 Frontier Science Center for Deep Ocean Multispheres and Earth System (FDOMES) and Physical Oceanography Laboratory/Key Laboratory of Ocean Observation and Information of Hainan Province, Sanya Oceanographic Institution, Ocean University of China, Qingdao/Sanya, China; 2 Laoshan Laboratory, Qingdao, China; 3 Department of Oceanography, University of Hawaii at Manoa, Honolulu, HI USA; 4 Center for Southern Hemisphere Oceans Research (CSHOR), CSIRO Oceans and Atmosphere, Hobart, Australia; 5 Joint Institute for Regional Earth System Science and Engineering, University of California, Los Angeles, CA USA; 6 State Key Laboratory of Numerical Modeling for Atmospheric Sciences and Geophysical Fluid Dynamics, Institute of Atmospheric Physics, Chinese Academy of Sciences, Beijing, China

**Keywords:** Physical oceanography, Fluid dynamics

## Abstract

Oceanic eddy-induced meridional heat transport (EHT) is an important process in the Southern Ocean heat budget, the variability of which significantly modulates global meridional overturning circulation (MOC) and Antarctic sea-ice extent. Although it is recognized that mesoscale eddies with scales of ~40–300 km greatly contribute to the EHT, the role of submesoscale eddies with scales of ~1–40 km remains unclear. Here, using two state-of-the-art high-resolution simulations (resolutions of 1/48° and 1/24°), we find that submesoscale eddies significantly enhance the total poleward EHT in the Southern Ocean with an enhancement percentage reaching 19–48% in the Antarctic Circumpolar Current band. By comparing the eddy energy budgets between the two simulations, we detect that the primary role of submesoscale eddies is to strengthen mesoscale eddies (and thus their heat transport capability) through inverse energy cascade rather than directly through submesoscale heat fluxes. Due to the submesoscale-mediated enhancement of mesoscale eddies in the 1/48° simulation, the clockwise upper cell and anti-clockwise lower cell of the residual-mean MOC in the Southern Ocean are weakened and strengthened, respectively. This finding identifies a potential route to improve the mesoscale parameterization in climate models for more accurate simulations of the MOC and sea ice variability in the Southern Ocean.

## Abstract (web summary)

Based on two high-resolution simulations, the authors find that submesoscale eddies significantly boost poleward oceanic heat transport in Antarctic waters by strengthening transport capability of mesoscale eddies through inverse energy cascade.

## Introduction

Satellite altimeter observations in the past three decades suggest that the Southern Ocean, the Antarctic Circumpolar Current (ACC) in particular, has the most widespread and energetic mesoscale eddies of the world ocean^1,2^. These mesoscale eddies with horizontal scales of 40–300 km are the oceanic analogs of atmospheric storms in dynamics and they have been demonstrated to play a leading role in the meridional transports of heat, salt, thickness, and other tracers in the Southern Ocean^2,3^. It is the eddy transport together with the competing wind-driven Ekman transport that jointly shapes the Southern Ocean’s residual-mean meridional overturning circulation (MOC)^4,5^. Given that large-scale fronts and their associated geostrophic currents (e.g., ACC) are nearly zonal in the Southern Ocean, mesoscale eddy-induced meridional heat transport (EHT) provides the key pathway to take warmer water from subtropical to subpolar regions^6,7^, whose variability substantially modulates the melting and re-freezing of sea ice around Antarctica^8–10^. Therefore, comprehensive knowledge of the EHT processes in the Southern Ocean is a prerequisite for understanding and predicting the variations of MOC and the Antarctic sea ice.

**Fig. 1:**
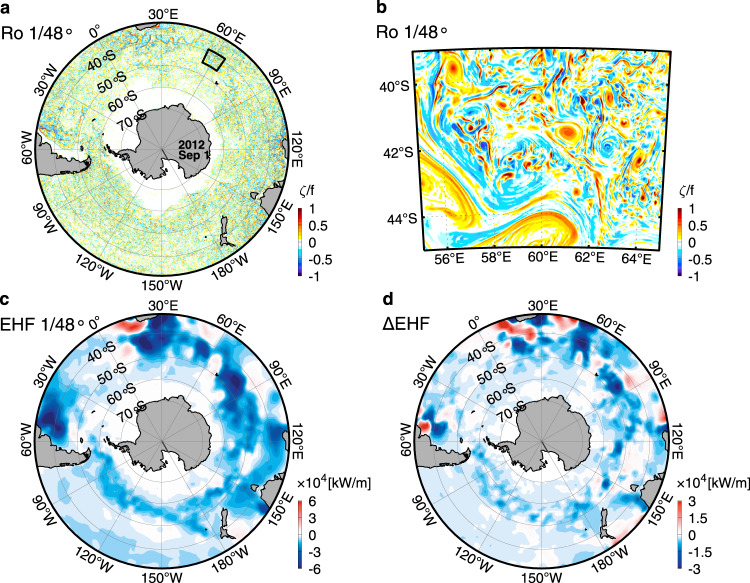
Distributions of Rossby number and eddy heat flux in the Southern Ocean. **a** Surface Rossby number on 1 September 2012 calculated using the relative vorticity divided by the planetary vorticity. **b** Zoom in on the black square in **a**. The distribution of Rossby number suggests that the Southern Ocean is abundant with both mesoscale and submesoscal eddies. **c** Vertically integrated total eddy heat flux (EHF) in the upper-1000 m averaged over the simulation period (positive for equatorward). Both the relative vorticity and the EHF in (**a**, **b**, **c**) are calculated based on the 1/48° simulation outputs. **d** Same as **c** but for the EHF difference between the 1/48° and 1/24° simulation-derived results (the former minus the latter). When submeoscale eddies are better resolved in the 1/48° simulation, magnitude of the EHF is significantly enhanced in the Southern Ocean compared with the 1/24° simulation.

In addition to active mesoscale eddies, recent high-resolution observations have revealed that the Southern Ocean is also abundant with strong submesoscale eddies (manifesting as fronts, filaments, and vortices) with 1–40 km horizontal scales^11–13^ (Fig. 1a, b). Because mesoscale strain fields provide favorable generation conditions for submesoscale eddies, their activities tend to be elevated at the periphery of mesoscale eddies^14^ (Fig. 1a, b). Compared with the quasi-geostrophic balanced mesoscale eddies with small Rossby numbers (i.e., a measure of relative importance of inertia and planetary rotation), submesoscale eddies have order one Rossby numbers and can induce much larger vertical velocities in the upper ocean due to their stronger ageostrophic nature^15–17^. Correspondingly, they result in large vertical heat fluxes with magnitude even comparable to air-sea heat fluxes^13,18^, leading to the possibility of significantly modulate the upper-layer heat budget of the Southern Ocean^18–20^.

Besides transporting heat vertically, the advection effect of submesoscale eddies can also generate meridional eddy heat fluxes (EHF) locally in a way similar to mesoscale eddies^21,22^. Furthermore, submesoscale eddies can interact with mesoscale eddies via a mutual energy exchange^23,24^ and may thus influence the mesoscale tracer transport indirectly^25^. Although it is known that submesoscale eddies potentially influence local divergence/convergence of heat fluxes, how and to what degree submesoscale eddies contribute to the total EHT in the Southern Ocean (i.e., the circumpolar integral of EHF) is unknown. This is at least partly due to the fact that resolving submesoscale eddies over the entire Southern Ocean is still beyond the capability of existing observations and most model simulations.

Here, we examine this issue by analyzing two state-of-the-art global ocean simulations, with one submesoscale permitting (1/48°) and the other only mesoscale resolving (1/24°). We find that submesoscale eddies effectively enhance the Southern Ocean total poleward EHT primarily by feeding mesoscale eddies via inverse energy cascade.

## Results

### Features of simulated eddies and eddy heat fluxes

In order to investigate the role of submesoscale eddies in the Southern Ocean EHT, the 1/48° and 1/24° global ocean simulations by the MITgcm (Massachusetts Institute of Technology general circulation model) are used here^18,26^. The 1/48° simulation has a horizontal resolution of 1–2 km in the Southern Ocean, which is not only able to fully resolve mesoscale eddies, but also can to a large degree resolve submesoscale eddies therein. By comparing the 1/48° simulation-derived large-scale and mesoscale quantities with the concurrent satellite observations, we find that the simulation has accurately reproduced the large-scale and mesoscale features in the Southern Ocean with respect to both patterns and magnitudes (Supplementary Figs. 1 and 2). In addition, the existing submesoscale-resolving observations have demonstrated that this simulation can reasonably capture the features of submesoscale eddies in the Southern Ocean^13,27^. The above analysis validates the performance of the 1/48° MITgcm simulation, which therefore gives us confidence to adopt it in this study. With respect to the 1/24° simulation, although it resolves mesoscale eddies in the Southern Ocean, it does not resolve mixed-layer instability, a leading generation mechanism of submesoscale eddies (Supplementary Fig. 3). The detailed information and validation of the model simulations can be found in Methods.

In Figs. 1a and 1b, we show the surface Rossby number on 1 September 2012 calculated using the 1/48° simulation (Methods). It can be seen that the Southern Ocean is indeed abundant with mesoscale eddies with scale of 40–300 km. They are characterized by *O*(0.1) Rossby numbers and typically occur in the form of coherent vortices and meanders. The activity of mesoscale eddies and thus their associated velocity and temperature anomalies are significantly elevated in the regions with strong large-scale currents such as the ACC, the Agulhas and its Return Currents, and the Brazil Current (Supplementary Fig. 2). Submesoscale eddies also occur ubiquitously in the Southern Ocean. Compared with the mesoscale eddies, the scale of submesoscale eddies is one order of magnitude smaller, and their Rossby numbers reach *O*(1). The submesoscale eddies tend to occur accompanying mesoscale eddies and their activities are particularly strong at the periphery of mesoscale eddies (Fig. 1b). The concurrence of mesoscale and submesoscale eddies suggests that they may interact and exchange energy with each other, which will be addressed in later sections.

Fig 1c shows the upper-1000 m vertically integrated mean EHF in the Southern Ocean calculated using the 1/48° simulation. The EHF here is the total quantity including contributions from both mesoscale and submesoscale eddies. It can be seen that the EHF is dominantly negative in the Southern Ocean, suggesting net southward (i.e., poleward) heat transports therein. The large positive EHF values mainly occur in the Agulhas leakage region southwest of Africa where detached mesoscale eddies (i.e., Agulhas rings) transport warm water northward into the Atlantic Ocean^28^.

The spatial pattern of the EHF magnitude agrees well with the eddy kinetic energy (EKE; Supplementary Fig. 4), both of which show elevated magnitudes in the ACC, the Agulhas Current, and the Brazil Current regions. Similar patterns between the EHF and EKE are consistent with the turbulent diffusion theory that the eddy diffusivity, the determinative factor of EHF, is proportional to the EKE^29,30^ (i.e., ${K}_{e}\propto \sqrt{EKE}\cdot {L}_{e}\propto EKE\cdot {T}_{e}$, where *K*_*e*_, *L*_*e*_, and *T*_*e*_ are diffusivity, horizontal length scale, and time scale of eddies, respectively). By comparing the EHFs obtained from the 1/48° and 1/24° simulations, we find that magnitude of the former is significantly larger than that of the latter. Specifically, their difference shows negative and positive values in the southward- and northward-EHF regions, respectively. In the large EHF regions, the magnitude of this EHF increase exceeds up to one-third of the EHF itself (Fig. 1c, d).

In order to examine the respective roles of mesoscale and submesoscale eddies in the total EHF and its enhancement, the mesoscale and submesoscale EHFs (EHF_me_ and EHF_sm_ hereafter) are calculated and compared (Supplementary Fig. 5). Spatially, both the EHF_me_ and EHF_sm_ show similar patterns to the total EHF. With respect to the magnitude, however, the EHF_me_ is close to the total EHF and the EHF_sm_ is one order of magnitude smaller. This result suggests that the total EHF in the Southern Ocean is primarily controlled by mesoscale eddies and the direct contribution of EHF_sm_ is limited. The above magnitude contrast can be explained by the mixing length theory^30^ because both the horizontal length and velocity scales of submesoscale eddies are much smaller than those of mesoscale eddies^14^.

### Submesoscale eddies enhance total eddy heat transport

**Fig. 2:**
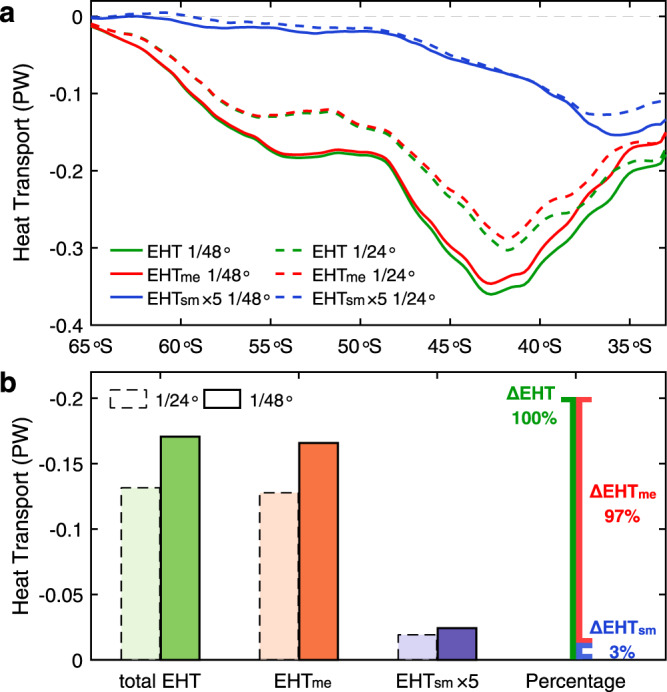
Latitudinal distributions and comparisons of eddy heat transports. **a** Green, red, and blue lines denote the total, mesoscale, and submesoscale eddy heat transports (EHTs), respectively. Results from the 1/48° and 1/24° simulations are denoted using solid and dashed lines, respectively. Note that the submesoscale EHT has been amplified by a factor of 5. **b** Averaged EHTs in the Antarctic Circumpolar Current band (i.e., 40–65 ^o^S) with the colors having the same meanings with **a**. Dark colors with solid-line edge and light colors with dashed-line edge denote results from the 1/48° and 1/24° simulations, respectively. The rightmost histogram shows the percentage partition of the increased mesoscale EHT (97%) and submesoscale EHT (3%) of the total EHT increase. Comparisons of different EHTs suggest that the better resolving of submesoscale eddies enhances not only the submesoscale EHT but also the mesoscale EHT. The enhanced total EHT is dominantly attributed to the mesoscale EHT enhancement.

Although submesoscale eddies’ direct contribution to the EHF is limited, it actually plays an important role in the total EHT in an indirect ways. Comparing the EHFs from the two different simulations suggests that better resolving of submeoscale eddies as in the 1/48° simulation not only better captures the EHF_sm_ itself but also enhances the EHF_me_. In particular, the EHF_me_ enhancement is one order of magnitude larger than the EHF_sm_, which therefore accounts for most of the total EHF enhancement (Fig. 1d and Supplementary Fig. 5c, d). Comparing the EHTs obtained through integrating the EHFs zonally give the same conclusion (Fig. 2a and Supplementary Fig. 6).

Specifically, in the ACC latitude band between 40–65 °S, the EHT_me_ (total EHT) increases by 20–45% (19–48%) in the 1/48° simulation compared with the 1/24° simulation. The ACC band-averaged EHTs of the 1/48° and 1/24° simulations are −0.17 and −0.13 PW, respectively (Fig. 2b), which means that including the effect of submesoscale eddies in the model can result in 30% more EHT southward across the ACC. With respect to the EHT_sm_, it on average increases by 26% in the ACC band. However, because of its small magnitude, this EHT_sm_ increase explains only 3% of the total EHT increase there. In other words, the remaining 97% increase of the total EHT is attributed to the strengthened EHT_me_. Below, we show that submesoscale eddies enhances the total EHT by energizing mesoscale eddies.

### Inverse energy cascade governs impact of submesoscale eddies

**Fig. 3:**
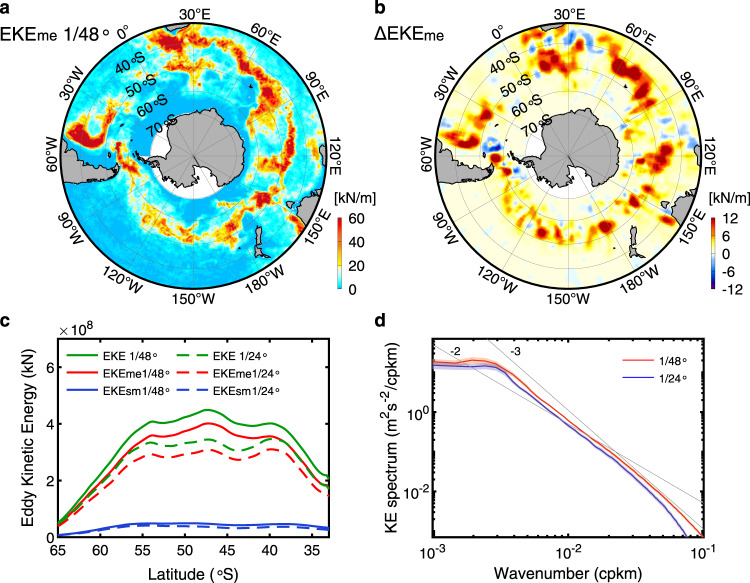
Comparisons of different eddy kinetic energy in the Southern Ocean. **a** Vertically integrated mesoscale eddy kinetic energy (EKE) in the upper 1000 m averaged over the simulation period from the 1/48° simulation outputs. **b** Same as **a** but for the difference between the 1/48° and 1/24° simulation-derived mesoscale EKE (the former minus the latter). **c** Latitudinal distributions of zonally integrated EKEs. Green, red, and blue lines denote the total, mesoscale, and submesoscale EKE, respectively. Results from the 1/48° and 1/24° simulations are denoted using solid and dashed lines, respectively. **d** Annually averaged kinetic energy spectra computed using horizontal velocities in the zonal 2000 km and meridional 1000 km box surrounding the center of the black square in Fig. 1a. The spectra are averaged over the upper 50 m. Red and blue lines denote results from the 1/48° and 1/24° simulations, respectively. Colored shadings represent 95% confidence intervals computed using bootstrap method. Gray lines denote the *k*^-2^ and *k*^-3^ scaling. The better resolving of submesoscale eddies in the 1/48° simulation significantly strengthens the EKE_me_. Although submesoscale EKE (EKE_sm_) is also strengthened, its increase is one order of magnitude smaller than the EKE_me_ increase.

Given that the magnitude of EHF is directly proportional to the EKE according to the turbulent diffusion theory^29,30^, we examine the simulated EKEs here to analyze the reason of the EHF and EHT enhancement in the 1/48° simulation. To better understand the respective contributions of mesoscale and submesoscale eddies, we apply the same comparison analysis as the EHF and EHT to the EKE (Fig. 3 and Supplementary Fig. 7). It shows that the mesoscale EKE (EKE_me_) is close to the total EKE and its magnitude is one order of magnitude larger than the submesoscale EKE (EKE_sm_, Fig. 3a and Supplementary Figs. 4 and 7a). This well explains why mesoscale eddies play a dominant role in the EHF.

Similar to the EHFs and EHTs, both EKE_me_ and EKE_sm_ are significantly strengthened in the 1/48° simulation compared with the 1/24° one (Fig. 3b, c and Supplementary Fig. 7b, c). The increases of both EKE_me_ and EKE_sm_ can also be seen from the wavenumber-dependent difference in the kinetic energy spectra calculated from the two simulations (Fig. 3d and Supplementary Fig. 8). As a result, the zonally integrated total EKE increases on average by 25% in the ACC band, for which 89% (11%) is explained by the EKE_me_ (EKE_sm_) increase. The above EKE increase and its partition (mesoscale vs. submesoscale) are generally consistent with those of the EHT increase. Our results demonstrate that the enhancement of EHT in the 1/48° simulation is primarily attributed to the increased EKE_me_ (i.e., the strengthened mesoscale eddies) while the direct role of the strengthened submesoscale eddies is limited.

**Fig. 4:**
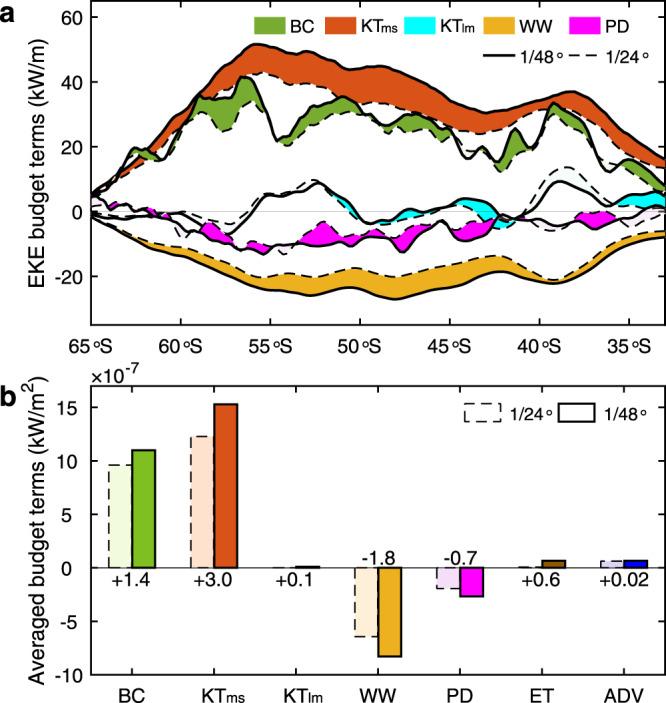
Mesoscale eddy kinetic energy budget of the Southern Ocean. **a** Latitudinal distributions of the zonally integrated mesoscale eddy kinetic energy (EKE_me_) budget terms, including baroclinic conversion from available potential energy (BC), kinetic energy transfer between mesoscale and submesoscale eddies (KT_ms_), kinetic energy transfer between mesoscale eddies and large-scale currents (KT_lm_), wind stress work (WW), and divergence of pressure work (PD). The detailed definitions of these budget terms can be found in Methods. Solid and dashed lines denote results from the 1/48° and 1/24° simulations, respectively. Differences between the solid and dashed lines are marked using fill colors with the dark and light ones denoting that the magnitudes are strengthened and weakened, respectively. **b** Spatially averaged EKE_me_ budget terms in the Antarctic Circumpolar Current band (i.e., 40–65 °S). Results from the 1/48° (1/24°) simulation are denoted by dark (light) colors with solid (dashed) edge lines. The abbreviations ET and ADV denote EKE_me_ tendency and advection of EKE_me_, respectively. Because of their small magnitudes, the ET and ADV are not plotted in **a**. Compared with the 1/24° simulation, the KT_ms_, which is the largest source term in the EKE_me_ budget, has the most notable increase in the 1/48° simulation. Although the source term BC has also increased in the 1/48° simulation, its increase magnitude is much smaller than the KT_ms_ and its effect is nearly offset by the increase of the two sink terms, i.e., WW and PD.

In order to explore the mechanisms of the EKE_me_ increase in the 1/48° simulation, we perform a budget analysis for the EKE_me_ and the results are shown in Fig. 4 (also Supplementary Fig. 9). Technical detail of this budget analysis is shown in Methods. We find that the baroclinic conversion from available potential energy and the mesoscale-submesoscale kinetic energy transfer (KT_ms_) are the two largest EKE_me_ source terms in both simulations. The large and positive baroclinic conversion is consistent with the knowledge that baroclinic instability is an important generation mechanism for mesoscale eddies in the Southern Ocean, which feeds the EKE_me_ through releasing available potential energy^31^. The positive KT_ms_ term means that mesoscale eddies obtain kinetic energy from submesoscale eddies through an upscale energy transfer^24^ (i.e., inverse cascade). Both the zonally integrated baroclinic conversion and KT_ms_ are prominently elevated in the ACC core band between 45–60 °S (Fig. 4a and Supplementary Fig. 9a–d), which well correspond to the energetic mesoscale and submesoscale eddies therein.

The work done by wind stress is an important EKE sink, known as “eddy killing” effect from winds^32,33^. In the 1/48° (1/24°) simulation, this damping effect dissipates 31% (29%) of the EKE_me_ obtained from the baroclinic conversion and KT_ms_ in the ACC band (Fig. 4b and Supplementary Table 1). The divergence of pressure work behaves as another EKE_me_ “sink” for the upper 1000 m, which exports the EKE_me_ to the deeper ocean. But its mean value is only one-third of the wind stress work in the ACC band. Except for the unknown parameterized dissipation term, the remaining three budget terms, including the EKE_me_ tendency, advection of EKE_me_, and kinetic energy transfer between large- and mesoscale processes, are negligible compared with the aforementioned three large terms when area mean over the ACC band is considered.

By comparing the EKE_me_ budget terms from the two simulations, we find that the most notable difference occurs for the KT_ms_ term. In the ACC band, its mean value increases by 25% in the 1/48° relative to the 1/24° simulation (Fig. 4b and Supplementary Table 1). This means that with an improved resolution of submesoscale eddies, the upscale kinetic energy transfer (or inverse cascade) from submesoscale to mesoscale eddies becomes more effective. Although the mean baroclinic conversion has also increased, the percentage of its increase is only 14% and its amount of increase is 54% smaller than that of the KT_ms_. The relatively small increase of baroclinic conversion is due to the fact that both the 1/48° and 1/24° simulations have sufficiently high resolutions to resolve the baroclinic instability process in the vast majority of the Southern Ocean, where the most unstable waves have wavelengths larger than 50 km^34^.

Corresponding to the strengthened mesoscale eddies (i.e., EKE_me_), the negative wind stress work that depends on the relative velocity between wind and sea surface current is also intensified. In addition, the negative pressure work divergence and the remaining three small positive terms also increase, but their increase amounts are one order of magnitude smaller than that of the KT_ms_. In the ACC band, the increase of the above two negative terms is nearly offset by the increase of baroclinic conversion and the three small positive terms, which therefore cancel each other out in the EKE_me_ budget (Supplementary Table 1). The above energetics analysis provides strong evidence that the strengthened EKE_me_ in the 1/48° simulation is primarily caused by the strengthened KT_ms_, i.e., inverse kinetic energy cascade by submesoscale eddies.

The above inverse energy cascade can also be inferred from the flattening of kinetic energy spectra in the meso- to submesoscale wavenumber range (i.e., length scales shorter than ~300 km) compared with the *k*^−3^ slope as in quasi-geostrophic turbulence theory^35^ (Fig. 3d, Supplementary Fig. 8). Specifically, due to the combination of inverse kinetic energy cascade by submesoscale eddies and forward enstrophy cascade by quasi-geostrophic turbulence, the spectral slope is between *k*^−2^ and *k*^−3^ (*k* is the horizontal wavenumber) for the 1/48° simulation, particularly in winter^36^. Note that although the 1/24° simulation cannot resolve the mixed-layer instability process, it can still resolve some submesoscale eddies generated by other mechanisms such as mesoscale strain-induced frontogenesis and horizontal shear instability^14^. As a result, submesoscale eddies and the associated inverse energy cascade are also observed in the 1/24° simulation, although their strengths are much weaker.

## Discussion

**Fig. 5:**
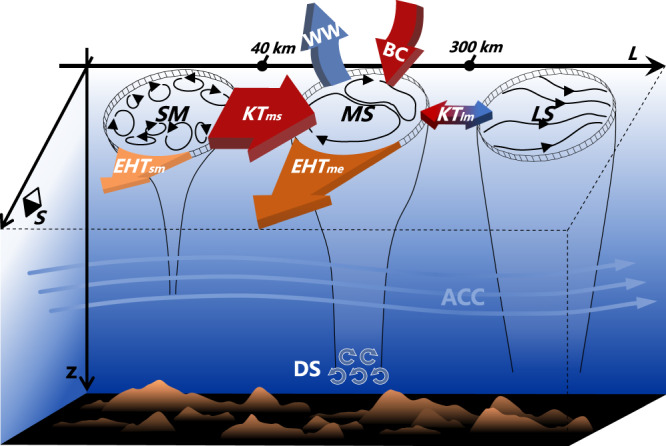
Schematic diagram of the eddy heat transport enhancement and its mechanism. The abbreviations SM, MS, LS represent submesoscale, mesoscale, and large-scale processes, respectively, whose separation scales are marked in the horizontal axis. The ACC is short for the Antarctic Circumpolar Current. The two orange arrows denote the poleward enhancement of mesoscale and submesoscale eddy heat transport (i.e., EHT_me_ and EHT_sm_) when submesoscale eddies are better resolved in the simulation. The other colored arrows denote the changes of the mesoscale eddy kinetic energy (EKE_me_) budget terms in the 1/48° relative to the 1/24° simulation, including kinetic energy transfer between mesoscale and submesoscale eddies (KT_ms_), baroclinic conversion from available potential energy (BC), wind stress work (WW), kinetic energy transfer between mesoscale eddies and large-scale currents (KT_lm_), and the dissipation term (DS). The red and blue arrows indicate the terms making positive and negative contributions to the EKE_me_ increase, respectively. When submesoscale eddies are better resolved in the 1/48° simulation, both the poleward submesoscale and mesoscale EHTs are significantly enhanced but the former is an order of magnitude smaller than the latter. The enhanced mesoscale EHT is attributed to the strengthened EKE_me_, which is primarily fed by the increase of KT_ms_, i.e., the strengthened upscale energy transfer from submesoscale to mesoscale eddies.

Our study has demonstrated that submesoscale eddies significantly enhance the poleward EHT in the Southern Ocean. Although submesoscale eddies can directly contribute to EHT, the EHT_sm_ itself is one order of magnitude smaller than the EHT_me_. The primary role of submesoscale eddies here is to enhance the mesoscale EHT (i.e., EHT_me_) by strengthening the mesoscale EKE via inverse energy cascade. A schematic diagram of this submesoscale inverse energy cascade-mediated EHT enhancement can be found in Fig. 5. Furthermore, we find that corresponding to the increased mesoscale EKE in the 1/48° simulation, the clockwise upper cell (anti-clockwise lower cell) of the Southern Ocean residual-mean MOC is weakened (strengthened) compared to that in the 1/24° simulation (Supplementary Fig. 10). Because the two simulations have the same atmospheric forcing and thus similar wind-driven mean overturning circulation, the above changes of MOC are primarily due to the stronger mesoscale eddy activities in the 1/48° simulation (i.e., EKE_me_), which induce an anti-clockwise overturning that counteracts the westerly wind-driven clockwise one^4^.

Because of their coarse resolutions, available satellite and in situ observations cannot resolve submesoscale eddies and the associated EHT process in the global ocean (or the entire Southern Ocean). However, the submesoscale inverse cascade that energizes mesoscale eddies as reported here has been demonstrated by localized in situ observations in different regions^17,37,38^. In addition, the submesoscale-mediated mesoscale eddy enhancement is supported by the comparison between the 1/48° simulation-derived EKE_me_ and those derived from another four widely used 1/10° or 1/12° eddy-resolving simulations (see Methods for model details). Partly due to the absence of submesoscale eddies and their inverse cascading effect, the zonally integrated EKE_me_ from these four eddy-resolving simulations on average accounts for 35–82% of that from the 1/48° simulation in the ACC band (Supplementary Fig. 11).

The previous study has proposed a parameterization of EHT_sm_ that depends only on the mixed-layer depth and cross-front buoyancy gradient, and which takes the form of an eddy-induced overturning streamfunction^39^. By comparing the “true” EHT_sm_ directly calculated from the 1/48° simulation with the parameterized EHT_sm_ based on the 1/24° simulation outputs (see the method in ref. ^40^), we find that although they have overall similar magnitudes, their spatial distributions are quite different (Supplementary Fig. 12). The inadequacy of this submesoscale parameterization is because it only considers the mixed-layer instability and not the other submesoscale generation mechanisms such as frontogenesis and horizontal shear instability^14^. The submesoscale-mediated enhancement of EHT through the inverse energy cascade, as described here, is not included in the prevailing parameterizations in present climate models^41^. Given that the EHT process significantly modulates the Southern Ocean heat content, the MOC, and the Antarctic sea-ice extent^10,42^, parameterizing the submesoscale inverse cascade and its EHT effect in future climate models is likely to improve the fidelity of simulations and their future projections. A feasible way for such parameterization is to extend the existing mesoscale eddy parameterizations to include the submesoscale inverse energy cascade^43^.

## Methods

### Model description

In order to investigate the EHT processes in the Southern Ocean, the MITgcm LLC4320 and LLC2160 simulations carried out on a latitude-longitude polar cap (LLC) grid are used^18,26^ (https://data.nas.nasa.gov/ecco/). The LLC4320 (LLC2160) simulation has a nominal horizontal resolution of 1/48° (1/24°), which ranges from 0.75 km (1.5 km) near Antarctica to 2.3 km (4.6 km) at the Equator. Both simulations have 90 levels in vertical direction, whose grid ranges from 1 m near the sea surface to ~47 m near the 1000 m depth and to ~480 m at the maximum depth of 6760 m. The two simulations have the same forcing fields and vertical mixing parameterizations.

Specifically, both of them are forced by six-hourly surface atmosphere fields from the 0.14° European Centre for Medium-Range Weather Forecasting (ECMWF) reanalysis product and the 16 most significant tidal constituents; their vertical mixing is parameterized using the widely used Richardson number-based K-profile parameterization scheme^44^. The model simulation outputs are stored hourly from 10 September, 2011 to 15 November, 2012 (14 months) and the daily averaged variables are used here (to remove the signals of tides and internal waves). For the 1/48° LLC4320 (1/24° LLC2160) simulation, its effective resolution ranges from 8 km to 4 km (16 km to 8 km) in the Southern Ocean between 30 °S and 65 °S^26^, which is smaller (larger) than the horizontal scale of the submesoscale mixed-layer instability at the same latitude^45^ (Supplementary Fig. 3). Therefore, the 1/48° simulation can to a large degree resolve the submesoscale eddies (particularly those generated through mixed-layer instability) in the Southern Ocean but the 1/24° one can hardly resolve them.

Given that horizontal scale of the baroclinic instability that generates mesoscale eddies is larger than 50 km in the Southern Ocean^34^, both the 1/48° and 1/24° simulations can well resolve the generation process of mesoscale eddies therein. The submesoscale permitting LLC4320 simulation has recently been applied to submesoscale studies in various regions of the world ocean^13,26,45–47^. More details about these two simulations can be found in Su et al.^18^. The model setup is available at https://github.com/MITgcm-contrib/llc_hires/.

In addition to the 1/48° and 1/24° MITgcm simulations, to highlight the role of submesoscale eddies, another four widely used eddy-resolving ocean circulation realistic simulation products are used for comparisons. These simulation products include the 1/12° HYCOM^48^, the 1/10° OFES^49^, the 1/10° BRAN^50^, and the 1/10° LICOM^51^. These four eddy-resolving simulations are different in several aspects, including the horizontal and vertical grids, the atmospheric forcing, as well as the subgrid parameterizations. The key similarity among them is that they can all resolve mesoscale eddies but cannot resolve submesoscale eddies in the Southern Ocean. Detailed configurations of these simulations can be found in references ^48–51^.

### Model validations

To evaluate the performance of the 1/48° MITgcm LLC4320 simulation in the Southern Ocean, we compare its 14-month mean large-scale and mesoscale quantities at sea surface with the results from the concurrent satellite altimeter (including sea surface height and absolute geostrophic velocity) and surface temperature data (Supplementary Figs. 1 and 2). Here, these two satellite data sets are obtained from AVISO (www.aviso.altimetry.fr/) and OISST (www.ncei.noaa.gov/data/), both of which have 1/4° spatial and daily temporal resolutions. For the large-scale process (see definition in the subsection “Isolation of mesoscale and submesoscale signals”), both the simulated mean kinetic energy and temperature show close distributions and magnitudes with the satellite observed ones (Supplementary Fig. 1). Similarly, the surface root-mean-squared mesoscale velocity and temperature anomalies (i.e., v’_me_ and T’_me_) in the simulation also display similar patterns and magnitudes to the observed results (Supplementary Fig. 2). For detailed model validation regarding the surface kinetic energy, one can refer to Qiu et al. (2018) (ref. ^46^) and Yu et al. (2019) (ref. ^52^) that compared the LLC4320 simulation with the satellite altimeter or surface drifter data.

In addition, the simulation-derived mesoscale EHF (Supplementary Fig. 5a) shows a similar pattern to the previous satellite observation-based results^53^, which provides another validation for the simulation results here. In contrast to the large- to mesoscale processes, submesoscale observations are not available on a large scale at present. However, the existing comparisons between the LLC4320 simulation and some localized submesoscale-resolving measurements in the Southern Ocean suggest that the LLC4320 can capture the basic features of submesoscale eddies^13,27^. The above comparisons and analysis demonstrate that the LLC4320 model can reasonably simulate the large-scale to submesoscale processes in the Southern Ocean and thus give us sufficient confidence to conduct this study.

### Calculation of Rossby number

The Rossby number (*Ro*), which is defined as the ratio of inertial to Coriolis terms in the horizontal momentum equations, is an important dimensionless parameter to characterize the relative importance of geostrophy. Typically, the Rossby numbers of mesoscale and submesoscale eddies are on the orders of 1 and 0.1, respectively^14^. Practically, the Rossby number can be quantified using the ratio between the vertical relative vorticity and the planetary vorticity, i.e.

1 $$Ro=\left(\frac{\partial v}{\partial x}-\frac{\partial u}{\partial y}\right)/f,$$

where *u*, *v*, and *f* are the zonal velocity, meridional velocity, and planetary vorticity, respectively. In order to depict the simulated features of mesoscale and submesoscale eddies, the Rossby number is calculated using the 1/48° daily averaged simulation outputs here.

### Isolation of mesoscale and submesoscale signals

In this study, we define the 14-month mean field with a horizontal scale larger than 4° as the large scale (i.e., ~190–385 km in the Southern Ocean between 30–65 °S). After removing the large-scale signals, we further define the perturbations with horizontal scales smaller and larger than 0.5° (i.e., ~24–48 km between 30–65 °S) as the submesoscale and mesoscale signals, respectively. In another word, the horizontal scale of mesoscale signals is between 0.5–4° (i.e., on average ~40–300 km). The isolation of different horizontal scales is completed using the corresponding low-, band-, or high-pass spatial filters. Here, the 0.5° separation scale between meso- and submesoscale is same as the LLC4320-based submesoscale study of Su et al. ^18^. The 4° is chosen as the upper bound of mesoscales because the influence size of an individual mesoscale eddy is generally smaller than 400 km in the Southern Ocean^2^. Note that all the above scale definitions and isolation are based on daily averaged data set, which has removed most of the internal waves (the inertial period, the upper bound of internal-wave period, is shorter than one day south of 30 °S). At present, the prevailing climate ocean models with a nominal resolution of ~100 km are only able to resolve processes with scale larger than ~400 km^54^. It means that even the largest mesoscale eddies (i.e., 4°) cannot be resolved by most of the present climate models.

### Calculation of EHF and EHT

The EHF is defined as

2 $${{{{{\bf{EH}}}}}}{{{{{{\bf{F}}}}}}}_{e}=\rho {C}_{p} < {T}_{e}^{{\prime} }{{{{{{\bf{v}}}}}}}_{e}^{{\prime} } > ,$$

where *ρ* is the sea water density, *C*_*p*_ = 4000 J kg^−1^°C^−1^ is the specific heat of sea water, and *T*′ and **v**′ are the temperature and velocity vector perturbations, respectively. Here, the subscript “e” denotes the mesoscale or submesoscale component, and the bracket denotes 4° or 0.5° spatial running mean based on the calculation object. Given that the **EHF**_*e*_ vector contains both rotational and divergent components but the former does not contribute to the net heat transport, we only retain the divergent **EHF**_*e*_ through applying the Helmholtz decomposition. Because we are concerned only with the vertically integrated meridional EHF, if not specified, the terms EHF_me_ and EHF_sm_ refer only to the meridional and divergent part of the corresponding **EHF**_*e*_ integrated over the upper 1000 m. The term EHF is used to denote the sum of EHF_me_ and EHF_sm_.

Based on the EHF_me_ and EHF_sm_, the corresponding EHT across each latitude is obtained through a circular zonal integral. Here, the reasons why we only focus on the upper 1000 m are twofold. First, the averaged EHT in the Southern Ocean accounts for ~80% of the total one integrated over the whole water column of the model (i.e., 6760 m). Second, including levels below 1000 m will result in large errors due to topography contamination in the spatial filters (particularly, for the EKE budget). In a similar way, the meridional large-scale mean heat transport can also be calculated.

### EKE budget analysis

The EKE_me_ budget is analyzed based on the vertically integrated EKE budget equation that simultaneously considers mesoscale’s interactions with both large scale and submesoscales. If we decompose the variable *A* into $A=\overline{A}+A^{\prime}+A^\ast$, where overbar, prime, and star denote large-, meso- and submesoscale component, respectively, the EKE budget equation for mesoscale can be written as follows^55^.

3 $$\begin{array}{rcl}\frac{\partial }{\partial t}\int EK{E}_{me}dz &=& -\int {{{{{\bf{v}}}}}}\cdot \nabla {EK}{{E}}_{{me}}dz-\int \nabla \cdot \overline{{{{{{\bf{v}}}}}}^{\prime} p^{\prime} }dz-\int g\overline{w^{\prime} \rho ^{\prime} }dz\\ & & -\int \rho \frac{\partial {\overline{u}}_{i}}{\partial {x}_{j}}\overline{{u}_{i}^{{\prime} }{u}_{j}^{{\prime}}} \,dz+\int \rho \overline{\frac{\partial {{u}}_{i}^{{\prime} }}{\partial {x}_{j}}{u}_{i}^{\ast }{u}_{j}^{\ast }}dz+\overline{{{{{{{\boldsymbol{\tau }}}}}}}_{{{{{{\bf{w}}}}}}}\cdot {{{{{{\bf{v}}}}}}}_{{{{{{\bf{top}}}}}}}^{{\prime} }}+DS\end{array}$$

Here, $EK{E}_{me}=\frac{1}{2}\rho ({u}^{{\prime} 2}+{v}^{{\prime} 2})$ is the mesoscale kinetic energy per unit volume, *p* is the pressure, *w* is the vertical velocity, and **V**_**top**_ (**τ**_**w**_) is the horizontal velocity (wind stress) at the sea surface; primes and stars denote the mesoscale and submesoscale anomalies, respectively, and overbars denote the large-scale (i.e., 4°) spatial running mean. Physically, the left-hand term is the EKE_me_ tendency (ET), and the right-hand terms from left to right represent EKE_me_ advection (ADV), divergence of mesoscale pressure work (PD), baroclinic conversion from available potential energy (BC), kinetic energy transfer between large- and mesoscales (KT_lm_), kinetic energy transfer between meso- and submesoscales (KT_ms_), mesoscale wind stress work (WW), and dissipation of EKE_me_ (DS). The DS is a generic dissipation term that includes contributions from bottom friction, turbulent viscosity, and pseudoviscosity in the model. Briefly, Eq. (3) can be rewritten as

4 $$ET=ADV+PD+BC+K{T}_{lm}+K{T}_{ms}+WW+DS.$$

Based on the EKE_me_ budget equation, each budget term is diagnosed using the outputs from both the 1/48° and 1/24° simulations. The vertical integration is performed between −1000 m and sea surface. Note that because the parameterized bottom drag and viscosities are not contained in the model outputs, we are unable to diagnose the DS term directly and can only regard it as a residue in the EKE_me_ budget. After each budget term is obtained, they are further averaged over the whole simulation period and spatially averaged (or integrated) along each latitude or over the ACC band.

### Supplementary information

Supplementary Information

## Data Availability

The LLC4320 and LLC2160 simulation data used in this study are available at https://data.nas.nasa.gov/ecco/data.php. The satellite altimeter data are downloaded from https://www.aviso.altimetry.fr/. The sea surface temperature data are downloaded from https://www.ncei.noaa.gov/data/. The data used for plotting the figures in the paper have been deposited in the Harvard Dataverse (10.7910/DVN/QWO84H).

## Code availability

The code of the LLC4320 and LLC2160 simulations is available at https://github.com/MITgcm-contrib/llc_hires/. Matlab 2018 was used to plot the figures.
