# Author Correction: Submesoscale inverse energy cascade enhances Southern Ocean eddy heat transport

**DOI:** 10.1038/s41467-023-44386-6

**Published:** 2023-12-18

**Authors:** Zhiwei Zhang, Yuelin Liu, Bo Qiu, Yiyong Luo, Wenju Cai, Qingguo Yuan, Yinxing Liu, Hong Zhang, Hailong Liu, Mingfang Miao, Jinchao Zhang, Wei Zhao, Jiwei Tian

**Affiliations:** 1 Frontier Science Center for Deep Ocean Multispheres and Earth System (FDOMES) and Physical Oceanography Laboratory/Key Laboratory of Ocean Observation and Information of Hainan Province, Sanya Oceanographic Institution, Ocean University of China, Qingdao/Sanya, China; 2 Laoshan Laboratory, Qingdao, China; 3 Department of Oceanography, University of Hawaii at Manoa, Honolulu, HI USA; 4 Center for Southern Hemisphere Oceans Research (CSHOR), CSIRO Oceans and Atmosphere, Hobart, Australia; 5 Joint Institute for Regional Earth System Science and Engineering, University of California, Los Angeles, CA USA; 6 State Key Laboratory of Numerical Modeling for Atmospheric Sciences and Geophysical Fluid Dynamics, Institute of Atmospheric Physics, Chinese Academy of Sciences, Beijing, China

**Keywords:** Physical oceanography, Fluid dynamics

Correction to: *Nature Communications* 10.1038/s41467-023-36991-2, published online 11 March 2023

The original version of this Article contained an error in the order of the project funding numbers in the Acknowledgements. The correct version states

‘This study was supported by the National Key Research and Development Program of China and the National Natural Science Foundation of China (2018YFA0605702, 42222601, 92258301, 42076004, 2022YFC3105003, 91958205, 91858203, 42230405). W.Z. is also supported by the 2022 Research Program of Sanya Yazhou Bay Science and Technology City (SKJC-2022-01-001). Z.Z. is also supported by the Fundamental Research Funds for the Central Universities (202041009) and the “Taishan” Talents program (tsqn202103032).’ in place of

‘This study was supported by the National Key Research and Development Program of China (2018YFA0605702, 2022YFC3105003), the National Natural Science Foundation of China (92258301, 42222601, 42076004, 91958205, 91858203, 42230405), the 2022 Research Program of Sanya Yazhou Bay Science and Technology City (SKJC-2022-01-001), and the Fundamental Research Funds for the Central Universities (202041009). Z.Z. is also supported by the “Taishan” Talents program (tsqn202103032).’.

This has been corrected in both the PDF and HTML versions of the Article.

