# Supplementary material for: Submesoscale inverse energy cascade enhances Southern Ocean eddy heat transport
Source: Nat Commun. 2023 Mar 11;14:1335. doi: 10.1038/s41467-023-36991-2 (PMC10008546; doi:10.1038/s41467-023-36991-2)
Supplement: Supplementary file 1 — Supplementary Information [file 41467_2023_36991_MOESM1_ESM.pdf]

Supplementary Information for

Submesoscale inverse energy cascade enhances Southern Ocean eddy heat transport

Zhiwei Zhang*, Yuelin Liu, Bo Qiu, Yiyong Luo, Wenju Cai, Qingguo Yuan, Yinxing Liu, Hong Zhang, Hailong Liu, Mingfang Miao, Jinchao Zhang, Wei Zhao*, Jiwei Tian*

*Corresponding authors. Email: zzw330@ouc.edu.cn; weizhao@ouc.edu.cn; tianjw@ouc.edu.cn

The supplementary information include:

Supplementary Figs. 1 to 12

Supplementary Table 1

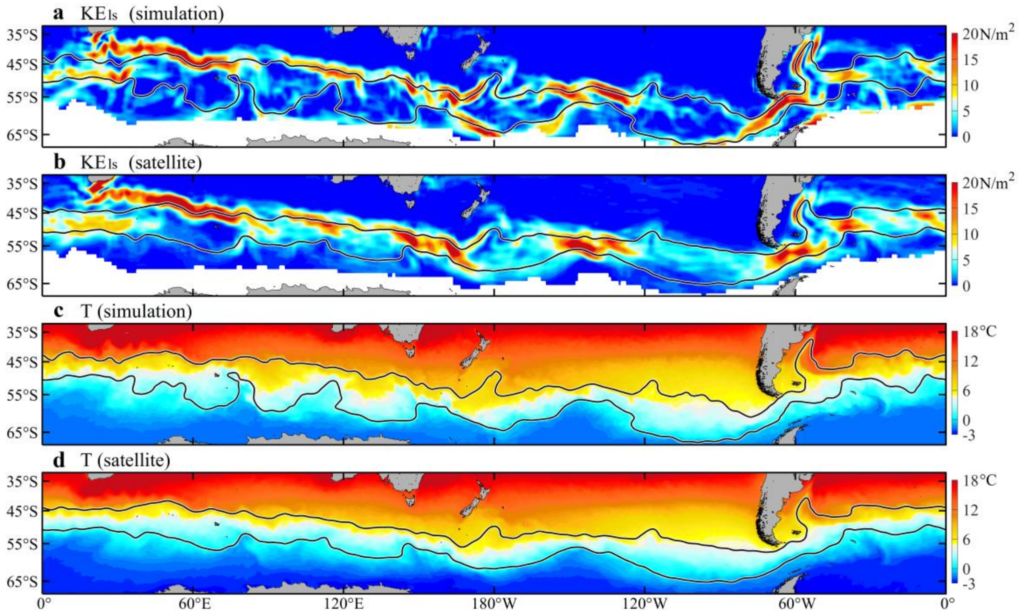

Supplementary Fig. 1 | Comparisons between the simulated and observed large-scale quantities. (**a**) Distribution of the large-scale surface geostrophic kinetic energy from the 1/48° simulation. (**b**) Same as (**a**) but for the satellite (altimeter) observation-derived result. Note that for a better comparison, the simulated results are not plotted in the regions without altimeter data (blank regions). (**c**, **d**) Same as (**a**, **b**) but for the large-scale surface temperature. Black lines in (**a, c**) denote the -0.8 and -0.1 m sea surface height contours, while those in (**b, d**) denote the -0.8 and 0.1 m contours.

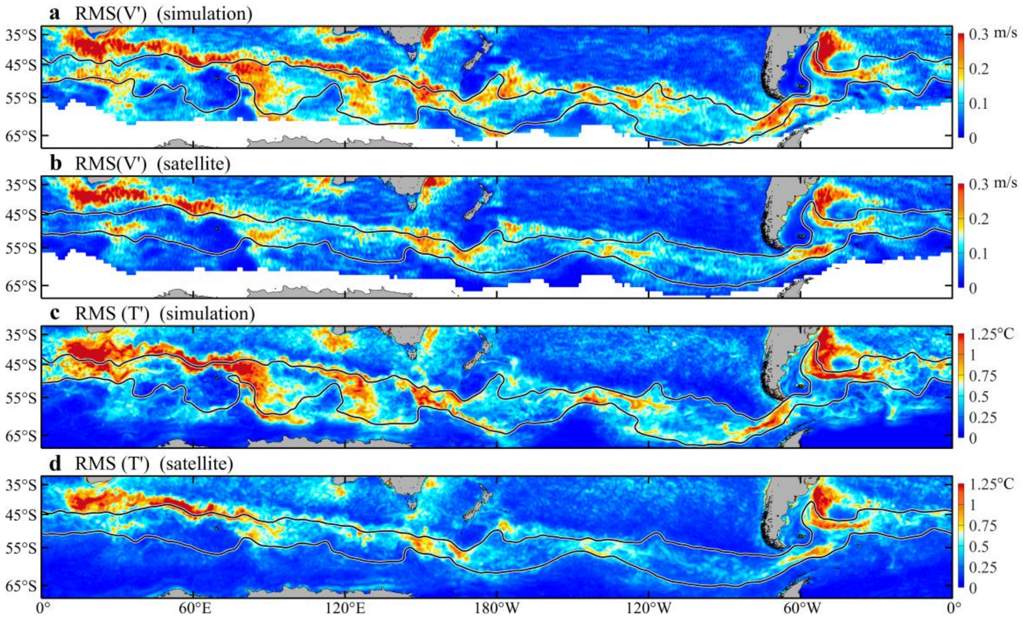

Supplementary Fig. 2 | Comparisons between the simulated and observed mesoscale quantities. (**a**) Distribution of the root-mean-squared mesoscale meridional velocity anomaly from the 1/48° simulation. (**b**) Same as (**a**) but for the satellite (altimeter) observation-derived result. Note that for a better comparison, the simulated results are not plotted in the regions without altimeter data (blank regions). (**c, d**) Same as (**a, b**) but for the root-mean-squared mesoscale temperature anomaly. Black lines have the same meanings with those in supplementary Fig. 1.

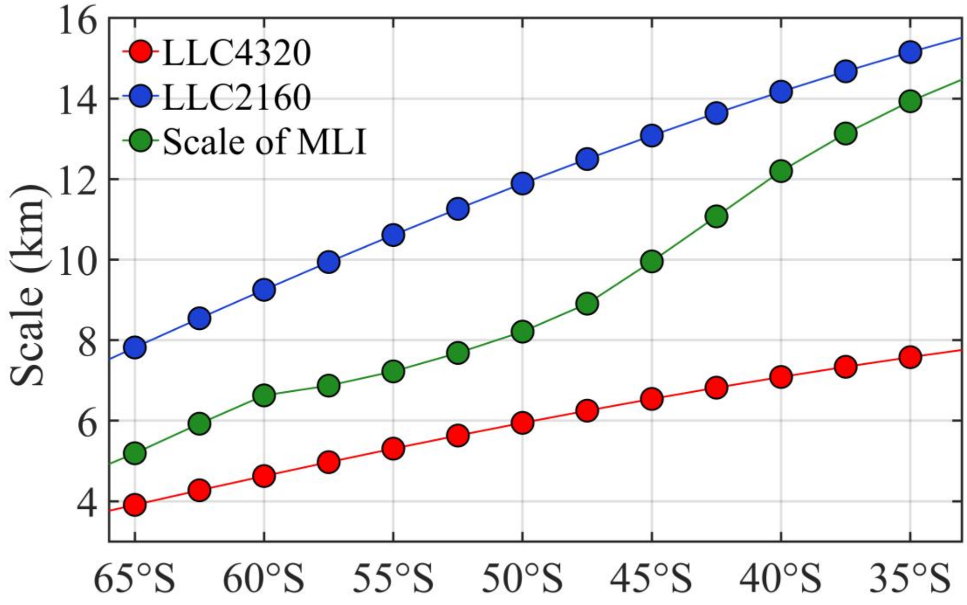

Supplementary Fig. 3 | Comparisons between the effective model resolutions and the scale of mixed-layer instability. Blue and red lines denote the effective resolutions of the 1/48° LLC4320 and the 1/24° LLC2160 simulations, respectively. Green line denotes the zonal median scale of mixed-layer instability (i.e. MLI) in winter as estimated by Dong et al[1].

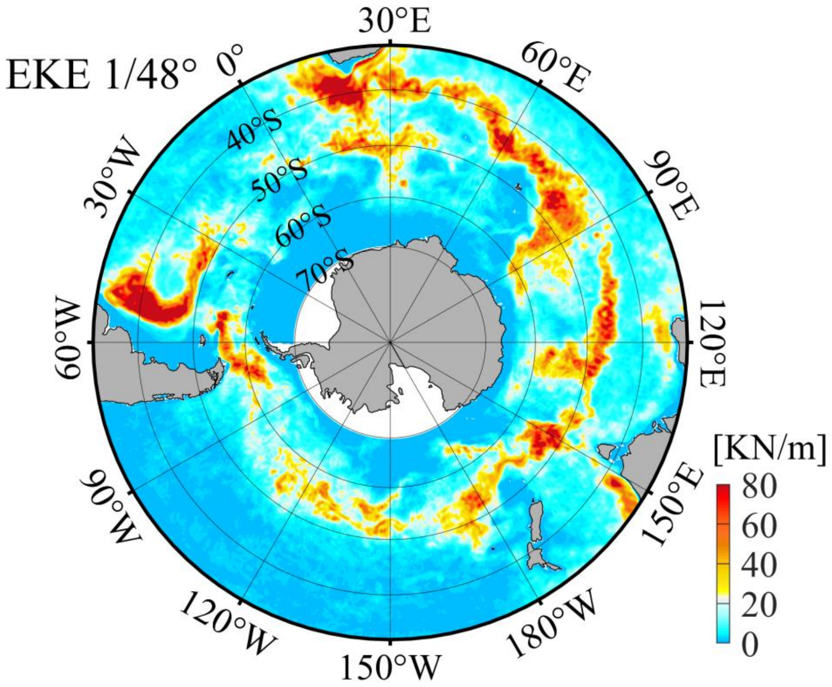

Supplementary Fig. 4 | Distribution of the vertically-integrated eddy kinetic energy from the 1/48° simulation. The eddy kinetic energy here is the total one including the contributions from both mesoscale and submesoscale eddies. The vertical integral is from -1000 m to sea surface.

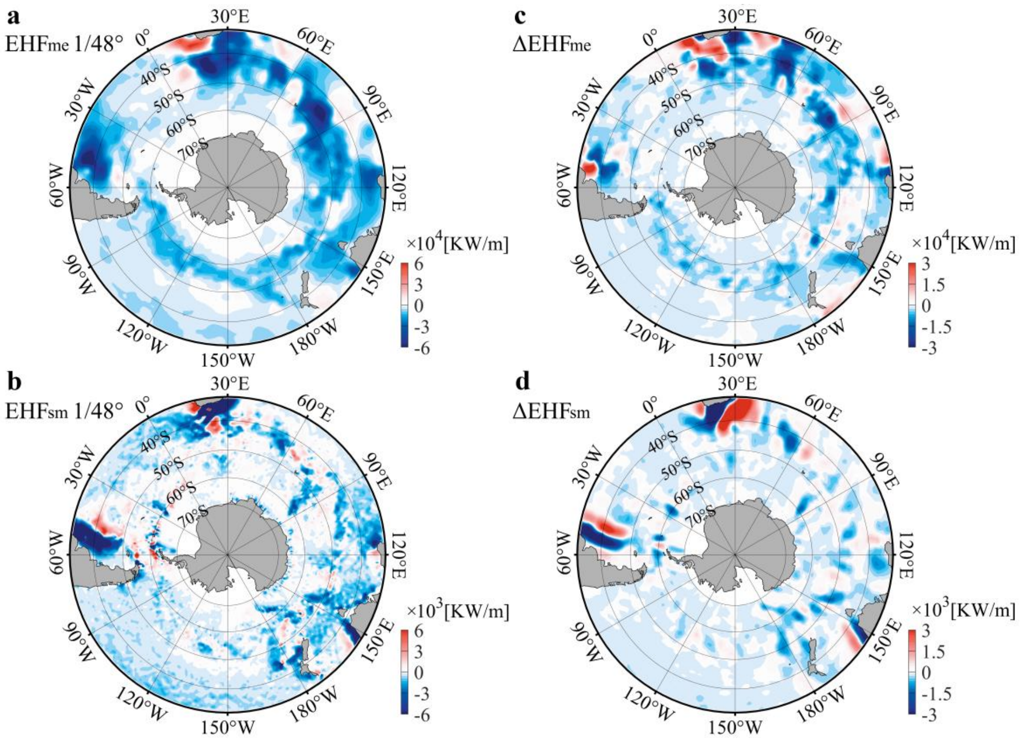

Supplementary Fig. 5 | Distributions of meso- and submesoscale eddy heat flux in the Southern Ocean. (**a**) Vertically-integrated mesoscale eddy heat flux (EHF) in the upper 1000 m averaged over the simulation period (positive for equatorward) from the 1/48° simulation outputs. (**b**) same as (**a**) but for the submesoscale EHF. (**c**, **d**) The differences between (**a**, **b**) and those from the 1/24° simulation.

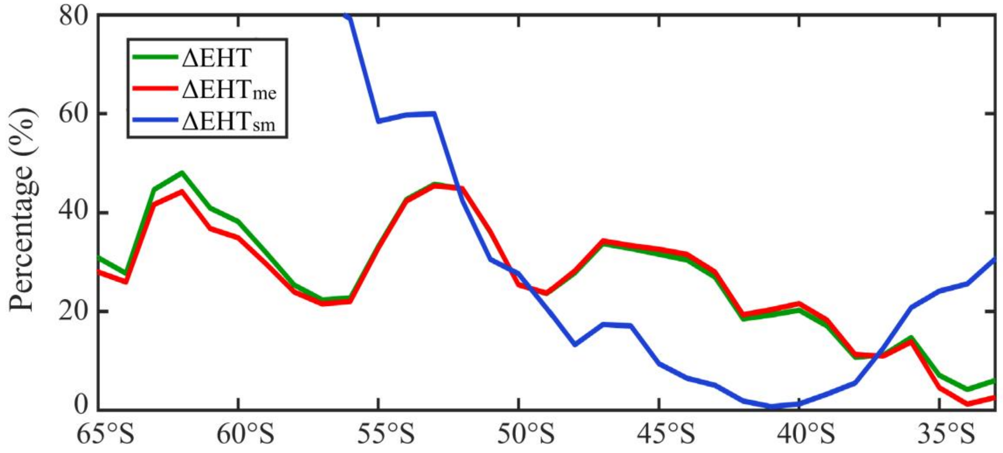

Supplementary Fig. 6 | Latitudinal distributions of the increase percentage of the eddy heat transports. Increase percentage of the eddy heat transports (EHTs) in the 1/48° simulation compared with those in the 1/24° simulation. Green, red, and blue lines denote the total, mesoscale, and submesoscale EHTs, respectively.

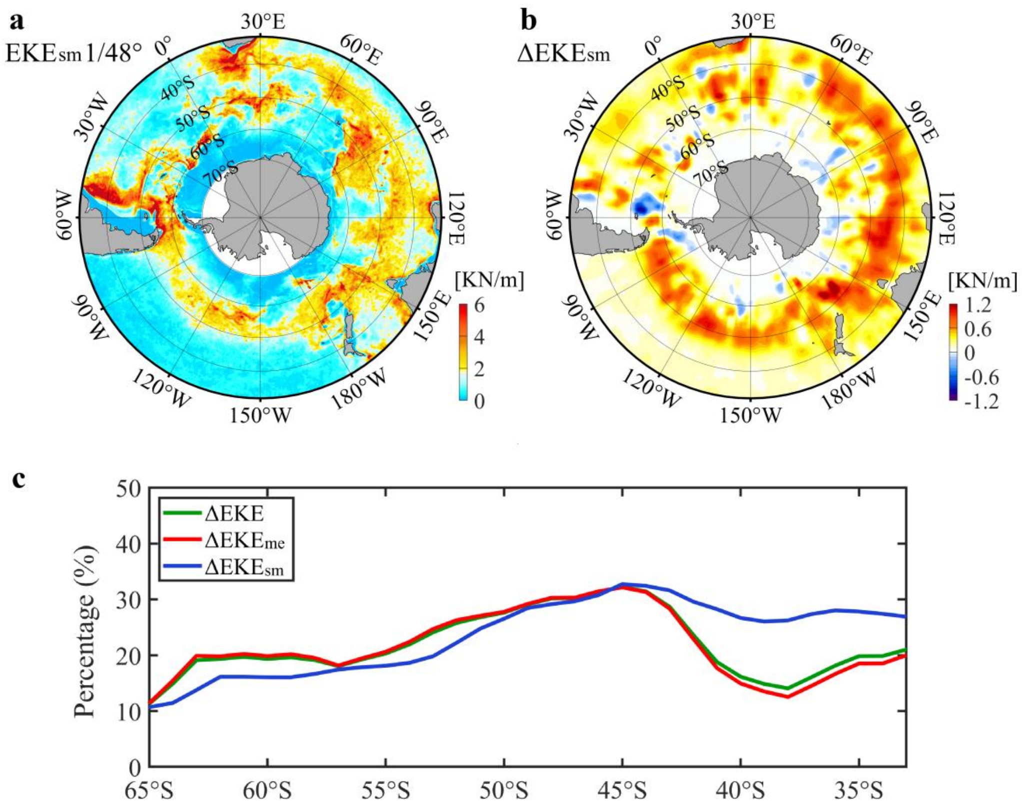

Supplementary Fig. 7 | Distributions of eddy kinetic energy and its increase in the Southern Ocean. (**a**) Vertically-integrated submesoscale eddy kinetic energy (EKE) in the upper 1000 m averaged over the simulation period. (**b**) Same as (**a**) but for the difference between the 1/48° and 1/24° simulation-derived submesoscale EKE (the former minus the latter). (**c**) Latitudinal distributions of the increase percentage of the different EKEs in the 1/48° simulation compared with those in the 1/24° simulation. Green, red, and blue lines denote the results for the total, mesoscale, and submesoscale EKE, respectively.

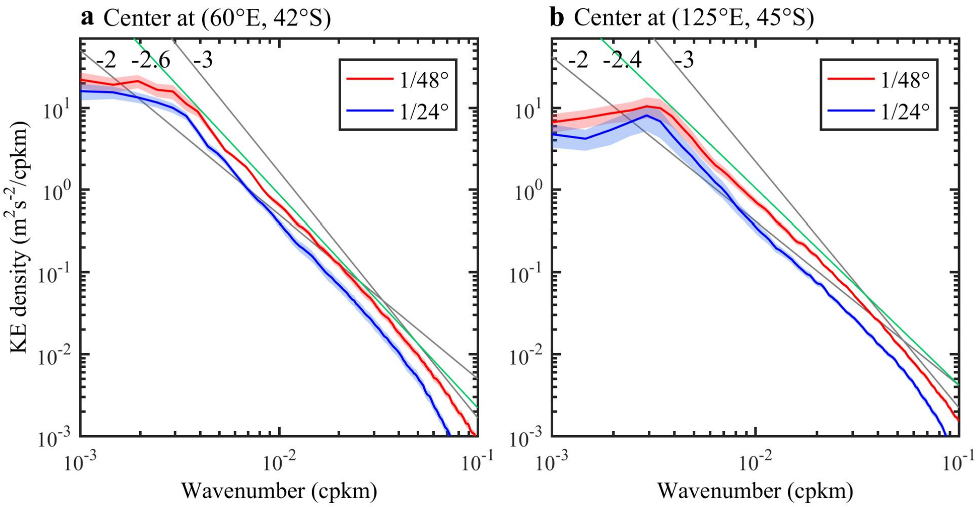

Supplementary Fig. 8 | Kinetic energy spectra in the Southern Ocean in winter. (**a**) Mean kinetic energy spectra in winter (July, August, and September) computed using horizontal velocities in the zonal 2000 km and meridional 1000 km box surrounding the center at (60 °E, 42 °S). The spectra are averaged over the upper 50 m. Red and blue lines denote results from the 1/48° and 1/24° simulations, respectively. Colored shadings represent 95% confidence intervals computed using bootstrap method. Gray lines denote the k^{-2} and k^{-3} scaling. (**b**) Same as (**a**) but for the results in the box with the center at (125 °E, 45 °S). Green line indicates the slope of the red line (i.e. result from the 1/48° simulation), which is -2.6 and -2.4 for (**a**) and (**b**), respectively.

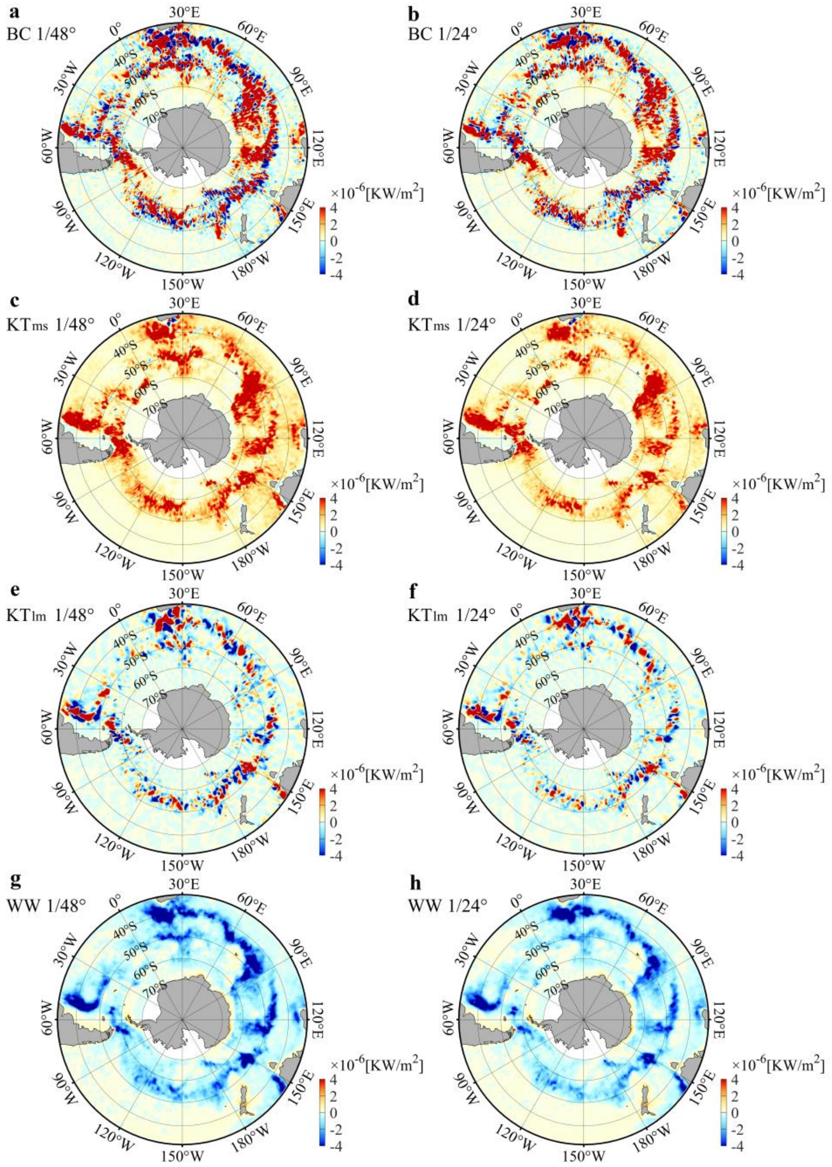

Supplementary Fig. 9 | Distributions of mesoscale eddy kinetic energy budget terms in the Southern Ocean. (**a**, **c**, **e**, **g**) show results of BC, KT_{ms}, KT_{lm}, and WW terms from the 1/48° simulation, respectively. (**b**, **d**, **f**, **h**) Same as (**a**, **c**, **e**, **g**) but for the results from the 1/24° simulation. Meanings of the abbreviations and detailed definitions of the budget terms can be found in Methods in the main text.

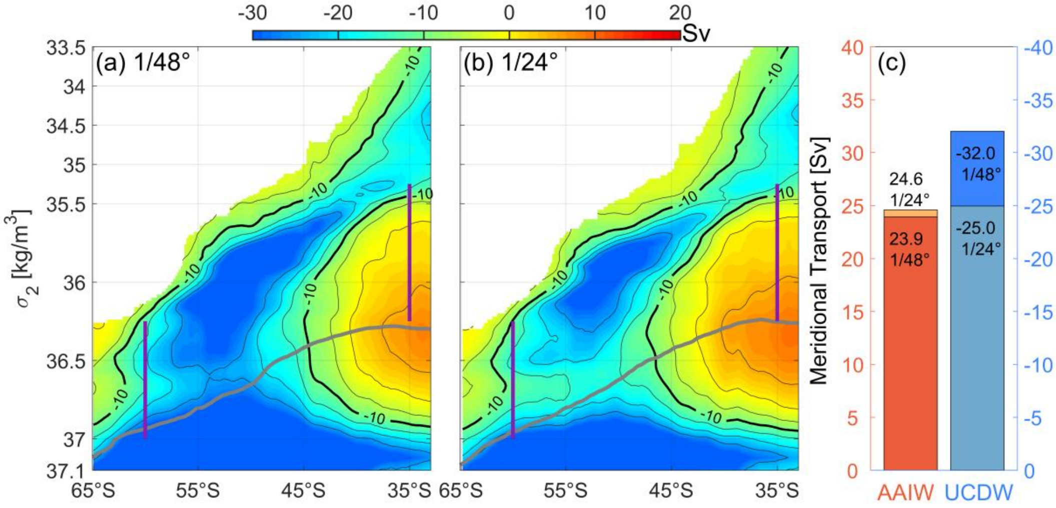

Supplementary Fig. 10 | Residual-mean meridional overturning circulation as a function of latitude and potential density. (**a**) and (**b**) are the 14 month-averaged results from the 1/48° and 1/24° simulations, respectively. Black lines are contours of the overturning stream function with an interval of 5 Sv (1 Sv = 10^6 m^3/s). The -10 Sv contour is highlighted using black thick line. Blue (yellow to orange) region denotes the anti-clockwise (clockwise) cell of the overturning circulation. The calculation is performed on the σ_2 coordinates (i.e. potential density referenced to -2000 m) using the same method with Ballarotta et al[2], in which the authors called such residual-mean overturning circulation as the "total" overturning circulation. Gray thick line denotes the mean potential density at -1000 m at each latitude. The two vertical purple lines denote the potential density ranges of the Upper Circumpolar Deep Water (UCDW; 36.25–37.00 kg/m^3) at 60 °S and the Antarctic Intermediate Water (AAIW; 35.25–36.25 kg/m^3) at 35 °S, respectively, which are used to compute meridional volume transports in (**c**). (**c**) Meridional volume transports of the UCDW and the AAIW across the two sections in (**a**, **b**), whose results are denoted by the right and left y axes, respectively (positive for northward).

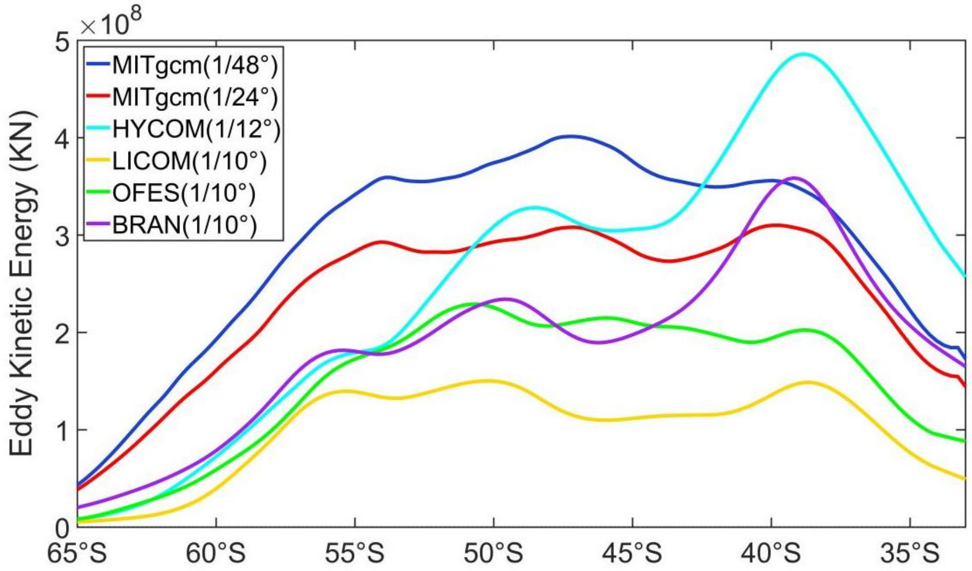

Supplementary Fig. 11 | Latitudinal distributions of mesoscale eddy kinetic energy derived from different simulations. Blue, red, cyan, yellow, green, and purple lines denote vertically-integrated mesoscale eddy kinetic energy in the upper 1000 m derived from the 1/48° MITgcm, 1/24° MITgcm, 1/12° HYCOM, 1/10° LICOM, 1/10° OFES, and 1/10° BRAN simulations, respectively. The Antarctic Circumpolar Current latitude band mentioned in the main text refers to 40–65 °S.

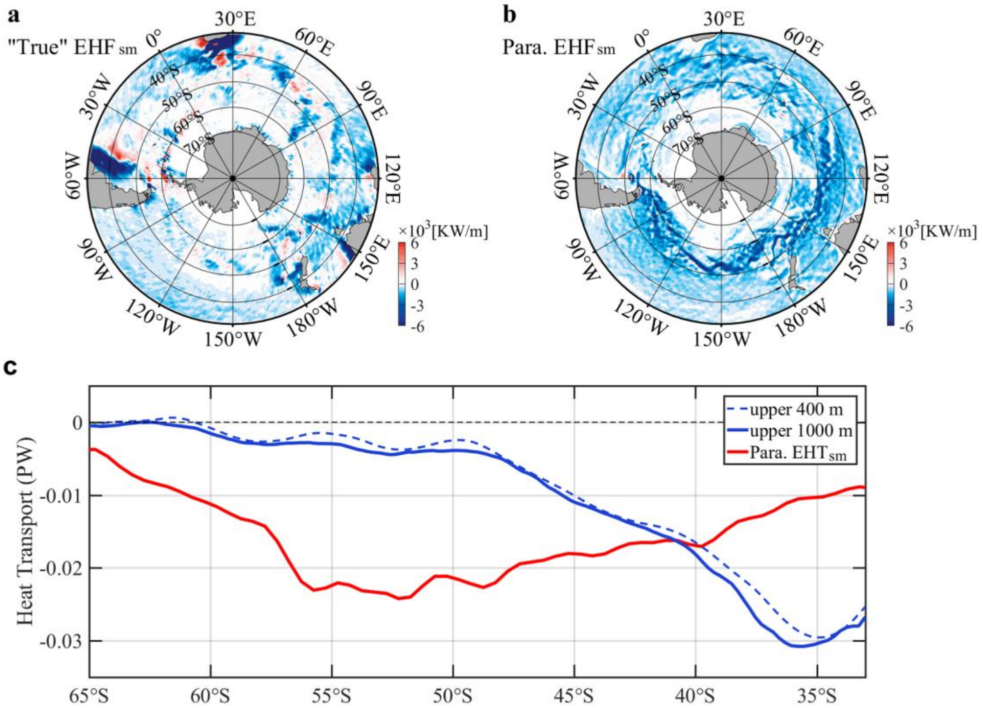

Supplementary Fig. 12 | Comparison between the "true" and parameterized submesoscale eddy heat flux and eddy heat transport. (**a**) The "true" submesoscale eddy heat flux (EHF) directly calculated from the 1/48° simulation. (**b**) The parameterized submesoscale EHF based on the 1/24° simulation outputs using the parameterization proposed by Fox-Kemper et al[3]. The calculation method of the parameterization is same with Calvert et al[4]. In the calculation, the 1/24° data are firstly averaged onto 0.5° grid to obtain the background fields. (**c**) Blue and red solid lines denote the "true" and parameterized eddy heat transport (EHT) integrated over the upper 1000 m, respectively. For a comparison, the "true" EHT integrated over the upper 400 m is shown using blue dashed line.

Supplementary Table 1 | Mesoscale eddy kinetic energy budget terms averaged in the Antarctic Circumpolar Current latitude band between 40–65 ^{o}S.

[10^{-8} KW/m^2]	1/24° Simulation	1/48° Simulation	Difference	Increase Ratio
**BC**	96.0	109.8	+13.8	14%
**KT_{ms}**	122.7	152.8	+30.1	25%
**KT_{lm}**	0.1	0.9	+0.8	833%
**WW**	-64.4	-82.4	-18.0	-28%
**PD**	-19.4	-26.7	-7.4	-38%
**Et**	0.7	6.7	+5.9	813%
**Adv**	6.3	6.6	+0.2	3%

Supplementary References

1. Dong, J., Fox-Kemper, B., Zhang, H. & Dong, C. The Scale of Submesoscale Baroclinic Instability Globally. *J. Phys. Oceanogr.* **50**, 2649-2667 (2020).
2. Ballarotta, M., Drijfhout, S., Kuhlbrodt, T., & Döös, K. The residual circulation of the Southern Ocean: Which spatio-temporal scales are needed?. *Ocean Model.* **64**, 46-55 (2013).
3. Fox-Kemper, B., Ferrari, R., & Hallberg, R. Parameterization of mixed layer eddies. Part I: Theory and diagnosis. *J. Phys. Oceanogr.* **38**, 1145-1165 (2008).
4. Calvert, D., Nurser, G., Bell, M. J., & Fox-Kemper, B. The impact of a parameterisation of submesoscale mixed layer eddies on mixed layer depths in the NEMO ocean model. *Ocean Model.* **154**, 101678 (2020).
